# Ultrasound-Guided Versus Blind Corticosteroid Injection for Trigger Finger: A Systematic Review

**DOI:** 10.1016/j.jhsg.2026.101175

**Published:** 2026-11-06

**Authors:** Aldana M. Alkuwari, Alreem Mohammad Al-khayarin, Khalifa Al Alawi, Fatima Al-jaber, Bara A. Shraim, Anwar Al-malki, Mohammed Muneer

**Affiliations:** ∗ College of Medicine, QU Health, Qatar University, Doha, Qatar; † Plastic Surgery Department, Hamad General Hospital, Hamad Medical Corporation, Doha, Qatar; ‡ Orthopedic Surgery Department, Hamad General Hospital, Hamad Medical Corporation, Doha, Qatar

**Keywords:** Blind injection, Corticosteroid injection, Stenosing tenosynovitis, Trigger finger, Ultrasound-guided injection

## Abstract

**Purpose:**

Trigger finger is the most common functional hand condition, affecting 3% of the general population and up to 17% of patients with diabetes. Corticosteroid injection is first-line treatment after failed conservative management and may be administered blindly or under ultrasound guidance (USG); however, the comparative clinical benefit of USG remains unclear.

**Methods:**

A systematic search was conducted across PubMed/MEDLINE, Embase, Scopus, Web of Science, and the Cochrane Library from inception to April 23, 2026, with an updated search through August 20, 2026, per PRISMA 2020 guidelines. Studies reporting outcomes following USG or blind corticosteroid injection for trigger finger were eligible. Risk of bias was assessed using the Cochrane Risk of Bias 2 tool for randomized trials and the Joanna Briggs Institute Critical Appraisal Checklist for Case Series for single-arm observational studies. This review was registered with the International Prospective Register of Systematic Reviews (PROSPERO; CRD420261378220). Because of substantial heterogeneity, a narrative synthesis was performed.

**Results:**

Six studies met the inclusion criteria, published between 2009 and 2026, encompassing 226 patients across the three studies reporting patient-level data and 223 fingers/digits across the three studies reporting digit-level data; these denominators are not directly summable given their differing units, with a female predominance and a mean age of 45–55 years. Ultrasound-guided injection was associated with faster early symptom resolution and lower visual analog scale/*Quick*DASH (Disabilities of the Arm, Shoulder, and Hand) scores in one study (*P* < .05 at 1–4 weeks), with this advantage attenuating by 12–24 weeks. The higher-quality randomized controlled trial demonstrated no significant difference in resolution rates between USG and blind injection (91% vs 83%, *P* > .05), a finding not consistently confirmed across comparative studies. Recurrence and surgical conversion findings were similarly inconsistent, and safety profiles were comparable between techniques, with no infections or tendon ruptures reported.

**Conclusions:**

Ultrasound-guided corticosteroid injection may offer modest early symptomatic benefit over blind injection in the treatment of trigger finger, but current evidence is limited by substantial risk of bias and does not support its routine use over blind injection as first-line therapy.

**Type of study/level of evidence:**

Therapeutic IV.

Trigger finger, stenosing flexor tenosynovitis, was first described by the French physician Alphonse Notta in 1850.^1^ It is considered to be the most common functional condition in hand surgery. It has a prevalence of 3% in the general population and up to 17% in the diabetic population.^2^, ^3^ It is an inflammatory condition characterized by thickening of the A1 pulley, which produces stenosis of the flexor tendon sheath and impairs tendon gliding. Clinically, it manifests as catching, clicking, or locking of the affected finger during flexion.^4^

Initial management of trigger finger is typically conservative, comprising physical therapy, splinting, and nonsteroidal anti-inflammatory drugs. Activity modification, which aims to reduce repetitive gripping and mechanical stress on the affected hand, forms an important component of this approach.^5^ In addition, a number of specific exercises have also been shown to relieve symptoms and preserve hand function, including tendon-gliding exercises, passive stretching, hook-fist exercises, and place-and-hold full-fist exercises.^6^, ^7^ Splinting may also be used to immobilize a specific joint (metacarpophalangeal, proximal interphalangeal, or distal interphalangeal joints), thereby limiting repetitive impingement of the flexor tendons against the stenosed pulley and allowing the associated inflammation to subside.^8^

If conservative management fails to improve symptoms, corticosteroid injection becomes the mainstay of management. A corticosteroid injection works by reducing inflammation, giving the tendon more freedom to glide. Its effectiveness has been shown in a randomized controlled trial, which compared 1 mL of triamcinolone acetonide with placebo (0.9% NaCl).^9^

Corticosteroid injection can be administered either blindly or under ultrasound guidance (USG). Several studies have compared the outcomes of these techniques, but the results are inconsistent.^10^, ^11^, ^12^ A systematic review and synthesis of the available evidence is therefore warranted to clarify whether ultrasound-guided corticosteroid injection offers clinically meaningful advantages over the conventional blind technique in the treatment of trigger finger.

## Materials and Methods

### Search strategy and information sources

This systematic review was conducted in accordance with the Preferred Reporting Items for Systematic Reviews and Meta-Analyses (PRISMA) guidelines (Fig. 1). The protocol was submitted to the International Prospective Register of Systematic Reviews (PROSPERO) before study screening and data extraction commenced and was formally registered on June 28, 2026 (CRD420261378220). Systematic and thorough research was conducted in five major electronic information databases, namely PubMed/MEDLINE, Scopus, Embase, Web of Science, and the Cochrane Library. The search was performed through the beginning of each database until April 23, 2026, with the search updated through August 20, 2026, to capture literature published in the interim. A complete, database-specific search strategy is provided in Supplementary Material S1 (available online on the Journal’s website at https://www.jhsgo.org). A search strategy was used that combined Medical Subject Headings (MeSH) terms and free-text keywords to ensure a high degree of both sensitivity and specificity. The Boolean string used was as follows: (“Trigger Finger” OR “Stenosing Tenosynovitis” OR “A1 Pulley”) AND (“Corticosteroid” OR “Steroid Injection” OR “Triamcinolone” OR “Methylprednisolone”) AND (“Ultrasound-Guided” OR “Ultrasonography” OR “USG”) AND (“Blind” OR “Anatomic Landmark” OR “Palpation-Guided”).

**Figure 1:**
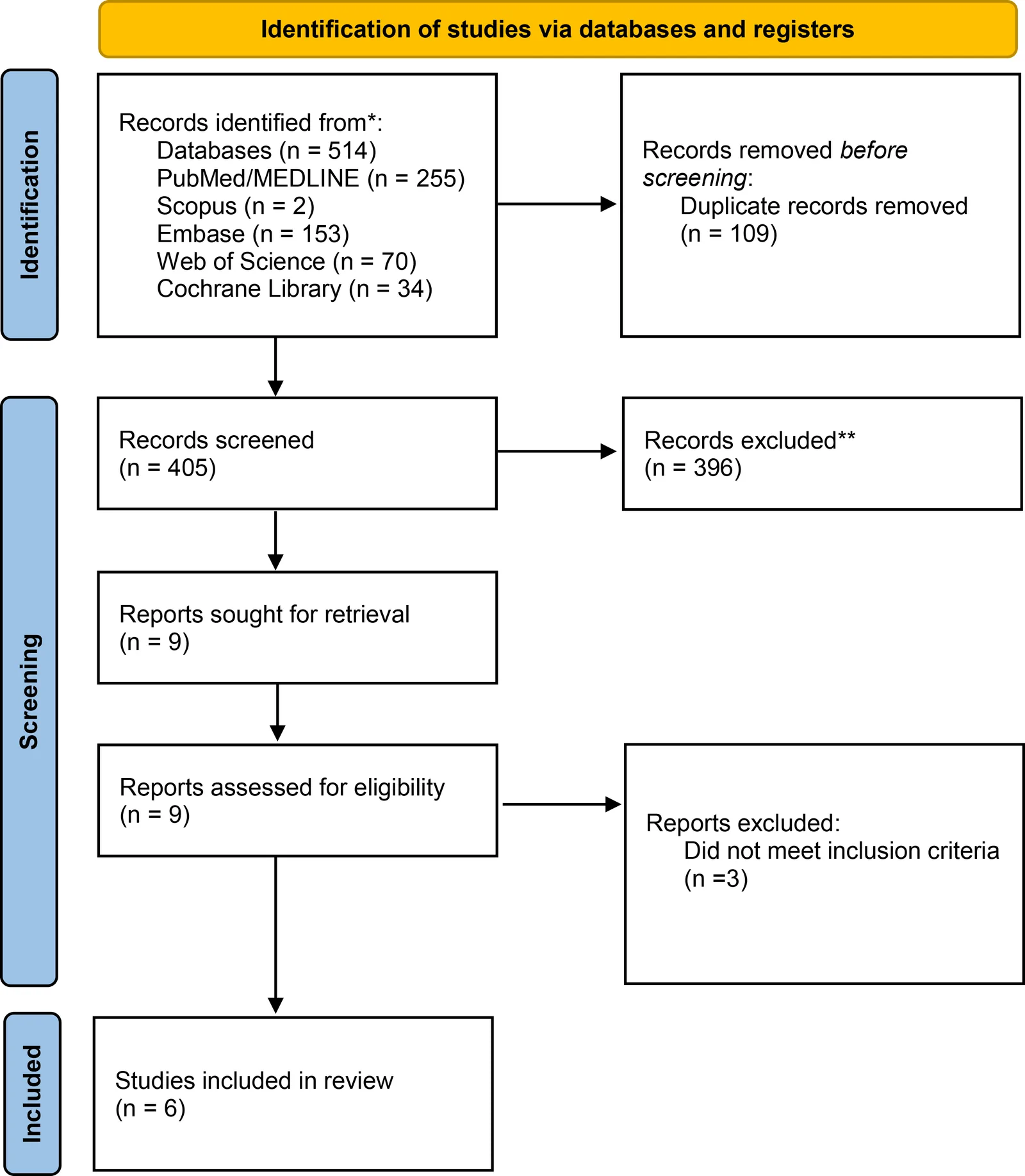
PRISMA diagram.

All identified primary studies, previous reviews, and relevant clinical guidelines were then reviewed for their reference lists to identify any potentially eligible studies not found through the initial database search. There was no language restriction at the beginning, but the final synthesis focused on studies with available English-language data.

### Study selection and eligibility criteria

Studies were selected using the PICOS (Population, Intervention, Comparison, Outcomes, and Study Design) framework predefined by the authors.

### Inclusion criteria

The following inclusion criteria were employed: Population (P), adult patients (age ≥18 years) diagnosed clinically with trigger finger (stenosing tenosynovitis) of any digit; Intervention (I), ultrasound-guided corticosteroid injection into the flexor tendon sheath or around the A1 pulley; Comparison (C), traditional blind corticosteroid injection using anatomical landmarks and palpation for the primary comparative synthesis. Studies without a concurrent comparator were eligible for inclusion as contextual evidence; Outcomes (O), primary outcomes recorded symptom resolution rates, rates of recurrence, and functional improvement (eg, visual analog scale [VAS] pain scores, DASH/*Quick*DASH [Disabilities of the Arm, Shoulder, and Hand] scores), and the secondary outcomes were complications (infection, tendon rupture, skin changes) and surgical conversion; Study Design (S), comparative clinical studies directly evaluating USG and blind corticosteroid injection were eligible for the primary comparative synthesis. Single-arm clinical studies evaluating either USG or blind corticosteroid injection were eligible as contextual evidence and were analyzed separately from the primary comparative synthesis.

### Exclusion criteria

The following were excluded for reasons of integrity and specificity of the surgical and clinical data: (1) nonhuman/nonclinical research, including animal models and cadaveric studies; (2) specific study types, including case reports, narrative reviews, meta-analyses, and existing systematic reviews, were excluded, although the reference lists of relevant reviews were screened for potentially eligible primary studies; (3) studies with anatomical mismatch, including studies involving corticosteroid injections for other joints or conditions (eg, de Quervain tenosynovitis, carpal tunnel syndrome, or general arthropathies); and (4) studies with a mismatch technique, including studies used to assess USG for a diagnostic purpose only or studies that used nonsteroid injectables (eg, platelet-rich plasma, hyaluronic acid) without a steroid comparative arm.

The selection process was performed in two phases. Two independent reviewers initially reviewed titles and abstracts for relevance. Second, full-text articles of potentially eligible studies were retrieved and assessed for inclusion/exclusion criteria. Disagreements between reviewers were settled by consensus or by obtaining input from a third senior researcher.

Eligible studies were stratified according to comparator type. The primary comparative synthesis included studies with concurrent USG and blind injection groups evaluated within the same study. Single-arm studies evaluating either USG or blind corticosteroid injection without a concurrent comparator were considered separately as contextual evidence. Given the different strength of inference provided by these study designs, contextual studies were not pooled or directly compared with the primary comparative synthesis. Where studies reported outcomes per digit rather than per patient, this was noted, and direct patient-level comparisons across studies were avoided.

### Data extraction and management

A standardized electronic extraction form was used to extract data from the included studies. The following data were taken: (1) study metadata, including lead author, year of publication, country of origin, and study design; (2) participant characteristics, including sample size (total and per group), sex distribution, affected digits, and age; (3) intervention details, including type and dose of corticosteroid injected, type of USG technique used (longitudinal vs transverse view), etc.; (4) outcome measures, including resolution, time points for follow-up (weeks/months), symptom resolution (n/%), *P* values for primary measures, VAS pain scores (mean ± standard deviation [SD]), functional scores (DASH/*Quick*DASH), and recurrence or conversion-to-surgery rates; and (5) safety data, including the incidence of skin depigmentation, fat atrophy, infection, and tendon rupture.

### Quality assessment and risk of bias

The methodological quality and risk of bias of included studies were assessed according to study design. Randomized controlled trials were evaluated using the Cochrane Risk of Bias 2 (RoB 2) tool, whereas single-arm observational studies were assessed using the Joanna Briggs Institute Critical Appraisal Checklist for Case Series. Assessments were performed independently by two reviewers, with disagreements resolved by consultation with a third reviewer.

### Data synthesis

A qualitative (narrative) synthesis was prioritized, as there was likely to be heterogeneity in the clinical and methodological characteristics of the included studies, including corticosteroid type, follow-up duration, and outcome reporting. Data were organized into outcome categories (resolution, function, and safety) for ease of comparison between the USG and blind techniques.

## Results

### Study selection

The first systematic search yielded 514 records across all databases (PubMed/MEDLINE n = 255; Embase n = 153; Web of Science n = 70; Cochrane Library n = 34; Scopus n = 2). After eliminating 109 duplicate records, 405 titles and abstracts were checked. During this stage, 396 records were excluded because they did not satisfy the basic eligibility criteria (mainly narrative reviews, case reports, or studies reporting on unrelated diseases).

Nine full-text reports were requested for retrieval and evaluated for their eligibility. Of these, three were removed because they did not meet the specific inclusion criteria. Based on these criteria, six studies were finally included in the final qualitative synthesis: Bodor and Flossman^13^, Faschingbauer et al^14^, Dala-Ali et al^15^, Cecen et al^12^, Tunçez et al^11^, and Abbaszadeh et al^10^ (Table 1, Table 2, Table 3, Table 4).^10^, ^11^, ^12^, ^13^, ^14^, ^15^

**Table 1:** Characteristics of Included Studies

Study	Year	Country	Design	Level of Evidence	Patients (n)	Fingers/Digits (n)	Comparator
Bodor & Flossman^13^ (2009)	2009	USA	Prospective single-arm study	IV	NR	50	None (USG single arm; historical benchmark cited)
Faschingbauer et al^14^ (2011)	2011	Austria	Prospective single-arm study	IV	NR	83	None (USG single arm; historical benchmark cited)
Dala-Ali et al^15^ (2012)	2012	UK	Retrospective single-arm study	IV	NR	90	None (blind injection single arm)
Cecen et al^12^ (2015)	2015	Turkey	Prospective randomized controlled trial	I	70	NR	USG vs blind (head-to-head)
Tunçez et al^11^ (2023)	2023	Turkey	Prospective randomized controlled trial	II	66	NR	USG vs blind (head-to-head)
Abbaszadeh et al^10^ (2026)	2026	Iran	Prospective randomized controlled trial	II	90	NR	USG vs blind (head-to-head)

NR, not reported; USG, ultrasound guidance.

**Table 2:** Summary of Primary Outcomes—Symptom Resolution, Recurrence, and Surgical Conversion

Study	USG Resolution	Blind Resolution	Reinjection USG	Reinjection Blind	Surgery (USG/Blind)	*P* Value (Resolution)
Bodor & Flossman^13^ (2009)	90% (1 y)	56% to 57% (historical benchmark; not concurrent)	≈4 fingers	N/A	0/N/A	<.01 vs historical benchmark
Faschingbauer et al^14^ (2011)	62% (>1 y)	N/A (single arm)	NR	N/A	N/A/17% (14/83)	N/A
Dala-Ali et al^15^ (2012)	N/A (blind-injection single arm)	66% (59/90 digits; up to 3 injections)	N/A	NR by injection round	N/A / 26 digits	N/A
Cecen et al^12^ (2015)	91% (32/35)	83% (29/35)	3/35 (9%)	6/35 (17%)	1/2 (P NS)	>.05 (NS)
Tunçez et al^11^ (2023)	91% (week 2)	86% (week 2)	6/32 (19%)	11/34 (32%)	2 / 2 (*P* = .164, NS)	NS at reported time points (severity, pain, *Quick*DASH)
Abbaszadeh et al^10^ (2026)	N/A (continuous outcomes only — see Table 3)	N/A (continuous outcomes only — see Table 3)	NR (repeat injection permitted at 1 week, not quantified by group)	NR (repeat injection permitted at 1 week, not quantified by group)	N/A (not reported)	NS at all time points (severity, pain, *Quick*DASH)

Note: Single-arm studies are presented as contextual evidence only. Historical benchmarks are not concurrent comparators and should not be interpreted as head-to-head comparisons.

N/A, not applicable; NR, not reported; NS, not significant.

**Table 3:** Summary of Secondary Outcomes—Functional Scores and Complications

Study	Time Point	VAS (USG vs Blind)	QuickDASH (USG vs Blind)	*P* Value	Complications
Cecen et al^12^ (2015)	Baseline → 6 months	4.74→0.54 vs 4.80→0.54	NR	NS (all time points)	None reported (either group)
Tunçez et al^11^ (2023)	1 week	0.30 ± 0.77 vs 0.83 ± 1.28	1.82 ± 3.40 vs 5.19 ± 7.77	*P* = .019/*P* = .017	—
Tunçez et al^11^ (2023)	4 weeks	0.06 ± 0.24 vs 0.39 ± 1.40	0.18 ± 0.73 vs 0.62 ± 1.59	*P* = .009/*P* = .005	2 transient neuropraxias (blind group)
Tunçez et al^11^ (2023)	12–24 weeks	Comparable between groups	Comparable between groups	NS	—
Abbaszadeh et al^10^ (2026)	Baseline → 12 weeks	2.13 ± 1.65→0.09 ± 0.35 (USG) vs 1.71 ± 1.72→0.04 ± 0.21 (blind)	30.8 ± 13.3→1.77 ± 4.2 (USG) vs 28.8 ± 14.7→0.86 ± 2.5 (blind)	NS at baseline, 6, and 12 weeks (*P* > .05)	Not reported by group

NR, not reported; NS, not significant; USG, ultrasound guidance; VAS, visual analog scale.

**Table 4:** Risk of Bias Summary

Study	Tool Used	Overall Judgment	Key Justification
Bodor & Flossman^13^ (2009)	JBI Critical Appraisal Checklist for Case Series	Met most criteria	Clear clinical/outcome reporting and follow-up; consecutive and complete inclusion could not be clearly established.
Faschingbauer et al^14^ (2011)	JBI Critical Appraisal Checklist for Case Series	Methodological uncertainty	Several aspects of participant selection, diagnostic assessment, and statistical methodology were insufficiently reported.
Dala-Ali et al^15^ (2012)	JBI Critical Appraisal Checklist for Case Series	Met all assessed criteria	Clearly defined eligibility criteria, standardized assessment and treatment methods, adequate follow-up, and appropriate statistical analysis.
Cecen et al^12^ (2015)	RoB 2	Some concerns	Randomized; lack of blinding raised concerns, while baseline characteristics and missing data were adequately handled.
Tunçez et al^11^ (2023)	RoB 2	High risk	Randomization method unreported; baseline imbalance in hypothyroidism (*P* = .006); participant/assessor blinding not feasible; subjective outcomes.
Abbaszadeh et al^10^ (2026)	RoB 2	Some concerns	Randomization method unreported; participant/assessor blinding not feasible; reinjection and complication outcomes not fully reported by group.

Note: JBI case-series appraisal is reported descriptively rather than as a numerical quality score. RoB 2 overall judgments are reported according to the Cochrane framework.

JBI, Joanna Briggs Institute; RoB 2, Cochrane risk of bias 2 tool.

### Study and participant characteristics

The studies ranged from level I randomized controlled trials to level IV retrospective reviews.

Regarding primary comparative synthesis, three prospective randomized controlled trials directly compared ultrasound-guided and blind corticosteroid injections: Cecen et al^12^, Tunçez et al^11^, and Abbaszadeh et al.^10^

Regarding contextual evidence from single-arm studies, Bodor and Flossman^13^ and Faschingbauer et al^14^ presented level IV evidence; the USG results were compared with historical benchmarks of blind injection in both studies. Dala-Ali et al^15^ provided retrospective single-arm evidence for blind corticosteroid injection.

Across the six included studies, 226 patients and 223 fingers/digits were accounted for; these totals use different denominators (patients vs digits) and are not directly summable.^10^, ^11^, ^12^, ^13^, ^14^, ^15^ The average age of the subjects was around 45–55 years; there was a marked female predominance (eg, 73% female in Tunçez et al). The lengths of the follow-up periods also varied widely, from 6 weeks to 3.5 years.

Because corticosteroid type, dosing, follow-up duration, and the definition of “resolution” varied across studies, the figures below are presented side by side for narrative comparison only and should not be interpreted as a pooled or statistically validated comparison.

### Symptom resolution rates

Symptom resolution was reported in five of the six included studies, although definitions of resolution and follow-up time points varied across studies. Abbaszadeh et al^10^ did not report a binary symptom resolution outcome and instead assessed changes in symptom severity, pain, and functional scores; therefore, its findings are presented separately below.

#### Ultrasound-guided group

The USG groups showed persistently high levels of symptom resolution. Cecen et al^12^ reported a 91% (32 out of 35) success rate with a single injection and 97% efficacy at 6 months. The results reported by Bodor and Flossman^13^ were 94% at 6 months and 90% at 1 year. The resolution rate was still at 71% after 3 years. The USG group had a 91% resolution rate by week 2, compared with 86% in the blind group by the same week, in the most recent study conducted by Tunçez et al.^11^ Over a 1-year period, Faschingbauer et al^14^ reported a 62% resolution rate, with findings such as tendinopathy (73%) and tenovaginitis (64%) affecting outcomes.

#### Blind injection group

The resolution rates for the blind groups tended to be lower or slower. Cecen et al^12^ reported a 6-month resolution rate of 83% (29 out of 35) after one injection and a 6-month cumulative efficacy of 94%. Dala-Ali et al^15^ found that the overall efficacy of blind injections was 66% (59 out of 90) after up to three injections, with the first injection successful in only 34% of cases. Tunçez et al^11^ noted a “slower resolution” in the blind group compared with USG. Bodor and Flossman^13^ had reported that their historical data showed only 56% to 57% success with blind injection.

#### Comparative significance

There was a statistically significant difference (*P* < .001) in resolution speed in favor of USG, according to Tunçez et al.^11^ The USG group had higher success after a single injection (91% vs 83%), but this difference was not statistically significant (*P* > .05), according to Cecen et al.^12^ However, when Bodor and Flossman^13^ compared it with historical blind injection cohorts, they found USG to be significantly superior (*P* < .01).

### Functional outcomes: Pain and disability scores

VAS and DASH/*Quick*DASH scores were used to assess pain and hand function.

#### VAS pain scores

Cecen et al^12^ reported that VAS scores improved from a baseline of 4.74 ± 1.25 to 0.54 ± 0.51 at 6 months in the USG group. Tunçez et al^11^ found that the USG group had significantly lower pain scores at 1 week (0.30 ± 0.77) and 4 weeks (0.06 ± 0.24) compared with the blind group (1 week: 0.83 ± 1.28; 4 weeks: 0.39 ± 1.40). The *P* values for VAS at 1 and 4 weeks were .019 and .009, respectively, indicating that USG was superior early on.

Abbaszadeh et al^10^ similarly found no significant difference in pain scores between groups at any time point: VAS improved from 2.13 ± 1.65 (USG) and 1.71 ± 1.72 (blind) at baseline to 0.09 ± 0.35 and 0.04 ± 0.21, respectively, at 12 weeks (*P* > .05 at baseline, 6, and 12 weeks), in contrast to the early advantage for USG reported by Tunçez et al.^11^

#### DASH/*Quick*DASH scores

The USG group had better functional recovery up to early time points in the study by Tunçez et al.^11^ The USG DASH scores were significantly lower at 1 week and 4 weeks (*P* = .017 and *P* = .005, respectively). Functional scores between the groups did not significantly differ at weeks 12 and 24.

In Abbaszadeh et al,^10^ *Quick*DASH scores improved from 30.8 ± 13.3 (USG) and 28.8 ± 14.7 (blind) at baseline to 1.77 ± 4.2 and 0.86 ± 2.5, respectively, at 12 weeks, with no significant between-group difference at any time point (*P* > .05).

### Recurrence and surgical conversion

The need for repeat injections or surgical release served as an indicator of treatment failure.

#### Repeat injections

Tunçez et al^11^ reported that 6 out of 32 (19%) of patients in the USG group required reinjection, compared with 11 out of 34 (32%) in the blind group (*P* < .001), indicating a significantly higher failure rate in the blind group. Cecen et al^12^ reported that 9% of the USG group and 17% of the blind group required an additional shot, all of whom were diabetic patients.

Abbaszadeh et al^10^ also permitted repeat injection at 1 week for patients without clinical improvement, but did not report reinjection counts stratified by group.

#### Conversion to surgery

The conversion rate to surgery was relatively low. Faschingbauer et al^14^ found that 14 out of 83 (17%) of their cohort went on to have surgery. Bodor and Flossman^13^ reported no cases of surgery in their USG group. Dala-Ali et al^15^ also observed that 26 digits in their retrospective blind injection group ended up needing open A1 pulley release.

Dala-Ali et al^15^ reported outcomes per digit (59 out of 90 digits) rather than per patient; their surgical conversion figure (26 digits) should not be directly compared with patient-level rates reported elsewhere.

### Injection accuracy

Precision of the positioning of the steroids was identified as one of the critical factors. Bodor and Flossman^13^ used USG visualization to verify 100% intrasheath accuracy. They cited previous studies indicating that blind injections are successful no more than 50% of the time. Likewise, for their USG arms, both Cecen et al^12^ and Tunçez et al^11^ used 100% direct visualization.

### Complications and adverse events

The safety findings were very similar across the included studies, and corticosteroid injection was generally a very safe procedure irrespective of the guidance method. Zero cases of infection were reported in any study. No cases of flexor tendon rupture were noted. Regarding skin changes, no occurrence of skin depigmentation or hypopigmentation was reported by Bodor and Flossman^13^, Cecen et al^12^, and Tunçez et al.^11^ No cases of subcutaneous fat atrophy were reported in the USG groups. Regarding neurological conditions, Tunçez et al^11^ found two cases (6%) of transient neuropraxia in the blind injection group that resolved spontaneously at week 2. There were no such events within the USG group.

### Risk of bias assessment

Methodological quality varied across the included studies.

Cecen et al^12^ was classified as having some concerns. The randomization was appropriate (low risk in domain 1); however, there was no blinding (high risk in domain 2), and participants and providers knew they were using USG equipment. Baseline characteristics and missing data were adequately handled.

Tunçez et al^11^ was classified as high risk overall via RoB 2. Although described as randomized, no randomization method (sequence generation or allocation concealment) was reported, and a significant baseline imbalance in hypothyroidism between groups (*P* = .006) raised concerns about domain 1. High-risk judgments in domains 2 and 4 reflect the inherent impossibility of blinding participants or outcome assessors given the visible use of ultrasound equipment. The study’s merits include 0% loss to follow-up and validated outcome measures.

The Joanna Briggs Institute appraisal demonstrated variable methodological quality among the three single-arm studies. Dala-Ali et al^15^ met all assessed criteria, with clearly defined eligibility criteria, standardized assessment and treatment methods, adequate follow-up, and appropriate statistical analysis. Bodor and Flossman^13^ met most criteria, although consecutive and complete participant inclusion could not be clearly established. Faschingbauer et al^14^ had greater methodological uncertainty because several aspects of participant selection, diagnostic assessment, and statistical methodology were insufficiently reported.

Abbaszadeh et al^10^ was assessed using RoB 2 and classified as having some concerns. The study was randomized, but the randomization method was not described. Participant/assessor blinding was not feasible given the visible ultrasound equipment, and reinjection/complication outcomes were not fully reported by group.

## Discussion

A systematic review was conducted to analyze the comparative effects of ultrasound-guided corticosteroid injection versus blind or palpation-guided injection techniques in adults with trigger finger. The findings suggest that ultrasound guidance may provide an early clinical advantage in symptom relief, pain reduction, and functional recovery; however, these benefits were not consistently demonstrated across studies and became less apparent at later follow-up. There was also no consistent evidence of reduced recurrence or conversion to surgical A1 pulley release.

The short-term gain reported with ultrasound assistance seems reasonable. Real-time visualization will enable the clinician to visualize the flexor tendon, tendon sheath, A1 pulley, and peritendinous neurovascular structures and to determine needle location and movement within the flexor sheath and surrounding anatomy as the needle enters the tendon sheath and as the injectate is distributed. This can help to get it closer to where you want it and to minimize any accidental intratendinous or superficial injections.^16^ Tunçez et al^11^ found that ultrasound-guided injection led to improvements in pain and functional outcomes in the first 4 weeks; moderate symptom improvement occurred sooner, and it was quicker to return to work. The findings of this study support the use of ultrasound guidance to shorten early recovery, rather than indicating a more successful outcome.

It is unclear how much its improved accuracy will be clinically meaningful. It is not automatically assumed that if a corticosteroid is correctly placed, there is a corresponding improvement in subsequent outcome because there is the possibility of a successful landmark-guided injection reducing the size of the A1 pulley and allowing the corticosteroid to diffuse around it. Thus, it is recommended that there be a difference between the accuracy of technical components and the clinical durability of the product, in the context of studies available in the package. Blind injection might be useful if landmarks are visible and if performed by an experienced clinician, whereas ultrasound may be more useful in challenging situations, as well as in cases of recurrence and/or failure of previous treatment.

Effective pain reduction is vital because pain, catching, and locking decrease hand use.^17^ Early VAS benefit with ultrasound guidance is applicable to patients seeking rapid functional restoration. However, the expected progression following corticosteroid injections should be taken into account. Yak et al^18^ reported that many patients improved within the first month, regardless of the type of guidance used. Thus, this does not mean short-term differences prove that blind injections do not work, but that they are observed alongside improvement in both groups.

Repeat injections have implications for the burden and cost of treatment. Dardas et al^19^ showed that a few may get successful results with second and third corticosteroid injections, but for many, further intervention and/or surgery is required. The same is true for national benchmark data, however, in that although initial injection achieves success in many patients, repeated injection increases success, but also increases progression to surgery.^20^ In some comparisons reported here, fewer repeat shots were required following ultrasound-guided treatment. This could be due to improved initial delivery, but it will require further substantiation from a randomized trial with reinjection criteria.

Less convincing evidence was available for important long-term outcomes (recurrence and conversion to surgery). Follow-up duration, disease severity, impact on the digit, symptom duration, and systemic diseases play a role in recurrence. Predictors of recurrence were explored by Grandizio et al^21^, who found no predictive link between injection technique and recurrence. Another study also reported recurrence following treatment with primary corticosteroid; many of these cases were within 2 years, highlighting the importance of longitudinal follow-up.^22^ Studies that last only a few months might therefore overestimate the potential for long-term success.

This pattern is reinforced by Abbaszadeh et al^10^, which found no significant difference between USG and blind injection in disease severity, pain, or *Quick*DASH scores at 6 or 12 weeks—notably, even earlier than the 12- to 24-week convergence observed by Tunçez et al^11^, suggesting that early USG advantages are not consistently reproducible across studies.

Diabetes should be addressed because it has been linked to trigger finger and can impact the effectiveness of treatment. Hollins et al^23^ concluded that HbA1c and diabetes chronicity had a greater effect on injection success than the mere presence of diabetes.^23^ These variables were reported inconsistently, and some may have contributed to heterogeneity.

Heterogeneity may also arise from the use of different corticosteroid preparations and dosages. Bookman et al^24^ discovered that corticosteroid dosage affects success rates, suggesting that medication protocol differences may influence outcomes between techniques. Ma et al^17^ found corticosteroid injection to be effective, but highlighted variability between randomized trials. Standardization of drug type, dosage, anesthetic, and injection technique is therefore important.

Both techniques appeared to be safe, with severe complications such as infection and tendon rupture occurring rarely. Ultrasound guidance may help reduce neurovascular injury through visualization, although complication rates are too low for strong comparative conclusions.^16^ Hansen et al^16^ also demonstrated that ultrasound can be integrated into clinical pathways with positive outcomes, provided practical and resource considerations are addressed.

Unfortunately, outcome definitions vary, follow-up is inconsistent, and operator experience differs across studies. Blinding was challenging, and there was performance and assessment bias. Luangjarmekorn et al^25^ showed that repeated-injection success is influenced by patient and disease features, further highlighting the importance of stratified analyses.

### Limitations

Several limitations should be considered when interpreting the findings of this review. The included studies varied in design and methodology, with three studies providing concurrent comparator groups. In addition, three additional single-arm studies were considered separately as contextual evidence. Variability was also observed in the reporting of study populations, with outcomes expressed by patient in some studies and by treated digit in others, limiting direct aggregation across all studies. In addition, blinding of participants and assessors was inherently challenging given the nature of ultrasound-guided interventions. Some clinical heterogeneity was present in corticosteroid type and dose, definitions of treatment resolution, and reporting of potentially relevant patient characteristics, including diabetes status and glycemic control. Finally, although some studies demonstrated faster early symptomatic improvement with ultrasound guidance, this finding was not consistent across all studies, and longer-term outcomes, including symptom resolution, recurrence, and progression to surgery, were generally comparable between approaches. These limitations highlight the need for larger, standardized comparative studies while allowing the available evidence to inform the relative benefits of ultrasound-guided and landmark-guided corticosteroid injection.

The findings of this systematic review suggest that USG corticosteroid injection may provide an early clinical advantage over conventional blind injection for the management of trigger finger, although this benefit was not consistently demonstrated across all included studies. Some studies reported earlier symptom resolution and improvements in pain and functional outcomes with USG injection. However, these findings were not consistently reproduced across the comparative evidence, and long-term outcomes were generally comparable between the two techniques. The safety profiles were also comparable between the two groups. No major adverse events were reported in either group, irrespective of guidance method. There are several limitations to the reported evidence, as it varied greatly in sample size, methodology, corticosteroid preparation, and outcome reporting. To confirm the results of the present systematic review, well-designed, adequately powered randomized controlled trials with standardized treatment protocols and longer follow-up are needed to determine whether the early benefits associated with USG injection translate into meaningful long-term clinical advantages.

## Conflicts of Interest

No benefits in any form have been received or will be received related directly to this article.
