# Supplementary material for: Ultrasound-Guided Versus Blind Corticosteroid Injection for Trigger Finger: A Systematic Review
Source: J Hand Surg Glob Online. 2026 Nov 6;9(1):101175. doi: 10.1016/j.jhsg.2026.101175 (PMC13769512; doi:10.1016/j.jhsg.2026.101175)
Supplement: Supplementary file 1 [file mmc1.docx]

**SEARCH STRATEGY**

**PubMed**

**Search A — Keyword Search:**

(

"trigger finger"[Title/Abstract] OR

"trigger digit"[Title/Abstract] OR

"stenosing tenosynovitis"[Title/Abstract] OR

"stenosing flexor tenosynovitis"[Title/Abstract] OR

"flexor tenosynovitis"[Title/Abstract] OR

"digital tenosynovitis"[Title/Abstract] OR

"A1 pulley"[Title/Abstract] OR

"flexor tendon sheath"[Title/Abstract]

)

AND

(

"ultrasound guided"[Title/Abstract] OR

"ultrasound-guided"[Title/Abstract] OR

"sonographic guidance"[Title/Abstract] OR

"ultrasonographic guidance"[Title/Abstract] OR

"ultrasound guidance"[Title/Abstract] OR

"image guided"[Title/Abstract] OR

"image-guided"[Title/Abstract] OR

"US-guided"[Title/Abstract] OR

"sonography guided"[Title/Abstract] OR

"real-time ultrasound"[Title/Abstract] OR

"point of care ultrasound"[Title/Abstract] OR

"POCUS"[Title/Abstract]

)

AND

(

"corticosteroid injection"[Title/Abstract] OR

"corticosteroid"[Title/Abstract] OR

"steroid injection"[Title/Abstract] OR

"cortisone injection"[Title/Abstract] OR

"blind injection"[Title/Abstract] OR

"palpation guided"[Title/Abstract] OR

"palpation-guided"[Title/Abstract] OR

"landmark guided"[Title/Abstract] OR

"landmark-guided"[Title/Abstract] OR

"conventional injection"[Title/Abstract] OR

"triamcinolone"[Title/Abstract] OR

"methylprednisolone"[Title/Abstract] OR

"betamethasone"[Title/Abstract] OR

"dexamethasone"[Title/Abstract] OR

"hydrocortisone"[Title/Abstract]

)

**Search B — MeSH Terms Search:**

(

"Trigger Finger Disorder"[MeSH] OR

"Tenosynovitis"[MeSH] OR

"Tendons"[MeSH]

)

AND

(

"Ultrasonography, Interventional"[MeSH] OR

"Ultrasonography"[MeSH] OR

"Point-of-Care Systems"[MeSH]

)

AND

(

"Injections"[MeSH] OR

"Injections, Intralesional"[MeSH] OR

"Adrenal Cortex Hormones"[MeSH] OR

"Glucocorticoids"[MeSH] OR

"Triamcinolone"[MeSH] OR

"Methylprednisolone"[MeSH] OR

"Betamethasone"[MeSH] OR

"Dexamethasone"[MeSH]

)

**EMBASE**

(

'trigger finger'/exp OR

'trigger digit':ab,ti OR

'stenosing tenosynovitis':ab,ti OR

'stenosing flexor tenosynovitis':ab,ti OR

'flexor tenosynovitis':ab,ti OR

'digital tenosynovitis':ab,ti OR

'A1 pulley':ab,ti OR

'flexor tendon sheath':ab,ti

)

AND

(

'ultrasound guided':ab,ti OR

'ultrasound-guided':ab,ti OR

'echography guided':ab,ti OR

'sonographic guidance':ab,ti OR

'ultrasonographic guidance':ab,ti OR

'ultrasound guidance':ab,ti OR

'image guided':ab,ti OR

'US-guided':ab,ti OR

'real-time ultrasound':ab,ti OR

'point of care ultrasound':ab,ti OR

'POCUS':ab,ti OR

'ultrasonography'/exp

)

AND

(

'corticosteroid injection':ab,ti OR

'steroid injection':ab,ti OR

'cortisone injection':ab,ti OR

'blind injection':ab,ti OR

'palpation guided':ab,ti OR

'palpation-guided':ab,ti OR

'landmark guided':ab,ti OR

'conventional injection':ab,ti OR

'triamcinolone'/exp OR

'methylprednisolone'/exp OR

'betamethasone'/exp OR

'dexamethasone'/exp OR

'glucocorticoid'/exp OR

'corticosteroid'/exp

)

**Cochrane CENTRAL**

(

"trigger finger" OR

"trigger digit" OR

"stenosing tenosynovitis" OR

"stenosing flexor tenosynovitis" OR

"flexor tenosynovitis" OR

"A1 pulley" OR

"flexor tendon sheath"

)

AND

(

"ultrasound guided" OR

"ultrasound-guided" OR

"sonographic guidance" OR

"ultrasonographic guidance" OR

"ultrasound guidance" OR

"image guided" OR

"image-guided" OR

"US-guided" OR

"real-time ultrasound" OR

"point of care ultrasound" OR

"POCUS"

)

AND

(

"corticosteroid injection" OR

"corticosteroid" OR

"steroid injection" OR

"cortisone injection" OR

"blind injection" OR

"palpation guided" OR

"palpation-guided" OR

"landmark guided" OR

"triamcinolone" OR

"methylprednisolone" OR

"betamethasone" OR

"dexamethasone" OR

"hydrocortisone"

)

**Web of Science**

"trigger finger" AND "ultrasound" AND "injection"

**Scopus**

( "trigger finger" OR "trigger digit" OR

"stenosing tenosynovitis" OR

"stenosing flexor tenosynovitis" OR

"flexor tenosynovitis" OR "A1 pulley" OR

"flexor tendon sheath" )

AND

( "ultrasound guided" OR "ultrasound-guided" OR

"sonographic guidance" OR

"ultrasonographic guidance" OR

"ultrasound guidance" OR "image guided" OR

"US-guided" OR "real-time ultrasound" OR

"point of care ultrasound" )

AND

( "corticosteroid injection" OR

"steroid injection" OR "blind injection" OR

"palpation guided" OR "palpation-guided" OR

"landmark guided" OR triamcinolone OR

methylprednisolone OR betamethasone OR

dexamethasone OR hydrocortisone )

**Documentation Table**

| **Database** | **Search Date** | **Search Used** | **Results (n)** |
| --- | --- | --- | --- |
| PubMed | 23/04/2026 | Full keyword string | n = 254 |
| EMBASE | 23/04/2026 | Full EMBASE string | n = 153 |
| Cochrane CENTRAL | 23/04/2026 | Full Cochrane string | n = 34 |
| Web of Science | 23/04/2026 | "trigger finger" AND "ultrasound" AND "injection" | n = 70 |
| Scopus | 23/04/2026 | Full Scopus string | n = 2 |
| TOTAL BEFORE DEDUPLICATION |  | All databases combined | n = 513 |
| DUPLICATES REMOVED |  | Removed via Rayyan | n = 109 |
| TOTAL AFTER DEDUPLICATION |  | Records for screening | n = 404 |
